# Plate coverage predicts failure for volarly unstable distal radius fractures with volar lunate facet fragments

**DOI:** 10.1051/sicotj/2020026

**Published:** 2020-07-27

**Authors:** Yuta Izawa, Yoshihiko Tsuchida, Kentaro Futamura, Hironori Ochi, Tomonori Baba

**Affiliations:** 1 Department of Trauma Center, Shonan Kamakura General Hospital 1370-1 Okamoto Kamakura-shi 247-8533 Kanagawa Japan; 2 Department of Orthopedic Surgery, Juntendo University School of Medicine 2-1-1 Hongo Bunkyo-ku 113-8431 Tokyo Japan

**Keywords:** Distal radius fractures, Volar lunate facet, Volar locking plate, Plate coverage

## Abstract

*Introduction*: Loss of reduction after operative fixation of volarly unstable distal radius fractures with a volar lunate facet fragment (VLF) is considered problematic because it results in carpal subluxation or dislocation and subsequent impaired function. We hypothesized that the indicator of loss of reduction of the VLF after fixation is plate coverage of the bony fragment. We investigated the relationship between the plate coverage of the VLF and loss of reduction after fixation, and calculated the plate coverage that was associated with failure of fixation of the VLF.

*Materials and methods*: We conducted a retrospective review. We included patients with surgically treated volarly unstable distal radius fractures with VLF with a volar locking plate who had a minimum follow-up of 6 months. A total of 33 patients (35 wrists) met criteria for inclusion into the study. The patients were divided into a displacement group and a non-displacement group. We calculated and compared longitudinal dimension and plate coverage of the VLF between the two groups to reveal the risk factors for loss of reduction.

*Results*: At final follow-up, 25 fractures maintained radiographic alignment and 10 (28.6%) lost reduction. Univariate analysis between the two groups showed that plate coverage against the transverse and longitudinal dimension of the VLF was correlated with loss of reduction after operative fixation. In multivariate logistic regression analysis, only plate coverage against the longitudinal dimension of the VLF remained a significant predictor of failure. With 64.7% as the cut-off point for plate coverage against the longitudinal dimension of the VLF, the sensitivity and specificity of the prediction were 96% and 80%, respectively.

*Conclusion*: The predictor of loss of reduction after fixation of volarly unstable distal radius fractures with a VLF was plate coverage against the longitudinal dimension of the VLF.

## Introduction

Loss of reduction after operative fixation of volarly unstable distal radius fractures with a volar lunate facet fragment (VLF) is considered problematic because it results in carpal subluxation or dislocation and subsequent impaired function, pain, limitation of range of motion, and posttraumatic arthritis [1, 2]. Previous studies have reported that the incidence of failure of fixation is 4–13% [2–4]. Several factors have been reported to contribute to this. Because the volar aspect of the lunate fossa bears more load than the scaphoid fossa when the wrist is in a position of function, a strong load is applied to the VLF. Furthermore, the short radiolunate ligament and volar distal radioulnar ligament, which stabilize the radiocarpal joint, attach to the VLF, so strong traction forces are applied to the fragment with motion of the wrist [1]. Therefore, the VLF requires rigid fixation. However, due to its unique anatomical feature of the ulnar volar margin of the lunate facet sloping volarly from proximal to distal and from radial to ulnar, the VLF may not be effectively supported by standard volar locking plate alone. Thus, a loss of reduction may occur when the fragment is small [1, 5].

Regarding the VLF size that can be treated with a standard volar plate, Beck et al. reported that the patients with volar shearing distal radius fractures with less than 15 mm of lunate facet available for fixation, or greater than 5 mm of initial lunate subsidence, are at risk of loss of reduction after fixation even if a standard volar plate is properly placed [2]. However, the size of the distal portion of the radius varies between individuals, so further investigation is necessary.

We hypothesized that the indicator of loss of reduction of the VLF after fixation is not the absolute value of the longitudinal dimension of the VLF, but plate coverage of the bony fragment. The purpose of our study was to investigate the relationship between the transverse and longitudinal dimension and plate coverage of the VLF and loss of reduction after fixation, and calculate the plate coverage that led to failure of fixation of the VLF.

## Materials and methods

This retrospective review was approved by the institutional review board, and informed consent was obtained from each patient.

Among 564 distal radius fractures that underwent operative fixation from August 2013 to September 2017, 40 wrists (7.1%) were volarly unstable fractures with separate scaphoid and lunate facet fragments. We included patients with surgically treated distal radius fractures with a volar locking plate who had a minimum follow-up of 6 months. A total of 33 patients (35 wrists) met criteria for inclusion into the study.

The patients included 13 men and 20 women who ranged in age from 20 to 91 years, with a mean age of 62 years. Twenty-four fractures resulted from a fall from standing height, five resulted from traffic accidents, four resulted from sports-related injuries, one resulted from a fall from height, and one occurred after a patient was hit by a car. Seventeen fractures were classified as type B3.3, 12 were classified as type C3.1, and 6 were classified as type C3.2 according to the AO/OTA comprehensive classification system.

Volar locking plates for distal radius fractures were placed using the AO/OTA comprehensive classification system with consideration of the size of the VLF. From August 2013 to October 2015, we placed a proximally placed volar locking plate when the longitudinal dimension of the VLF was larger than 8 mm and a distally placed volar locking plate when the longitudinal dimension of the VLF was less than 8 mm. As a result, 19 wrists were treated with a proximally placed volar locking plate and no wrists were treated with a distally placed volar locking plate. From October 2015 to September 2017, based on Beck’s report, for volar shearing fractures with separate scaphoid and lunate facet fragments we placed a proximally placed volar locking plate when the longitudinal dimension of the VLF was larger than 15 mm and placed a distally placed volar locking plate when the longitudinal dimension of the VLF was less than 15 mm. As a result, 6 wrists were treated with a proximally placed volar locking plate and 10 wrists were treated with a distally placed volar locking plate.

Loss of reduction was defined as greater than 4 mm of difference in carpal translation from the first postoperative radiograph to the final follow-up [2]. Carpal translation was defined as the distance between a line drawn collinear with the volar shaft of the radius and the center of the capitate.

The patients were divided into a displacement group (group D) and a non-displacement group (group N). Demographic data (age, sex, mechanism of injury, history of smoking, history of diabetes) and the AO/OTA classification were compared between these two groups (Table 1).

**Table 1:** Patient characteristics.

	Group D (*n* = 10)	Group N (*n* = 25)	*p* value
Age, years (mean [*SD*])	61.5 (20)	63.6 (18)	0.776
Range	23–87	20–91	
Sex, *n*			0.703
Female	7	14	
Male	3	11	
Mechanism, *n*			0.321
Low	10	20	
High	0	5	
Smoking, *n*	1	3	1
Diabetes, *n*	1	4	1
AO/OTA classification, *n*			0.367
B3.3	6	11	
C3.1	3	9	
C3.2	1	5	

**Figure 1:**
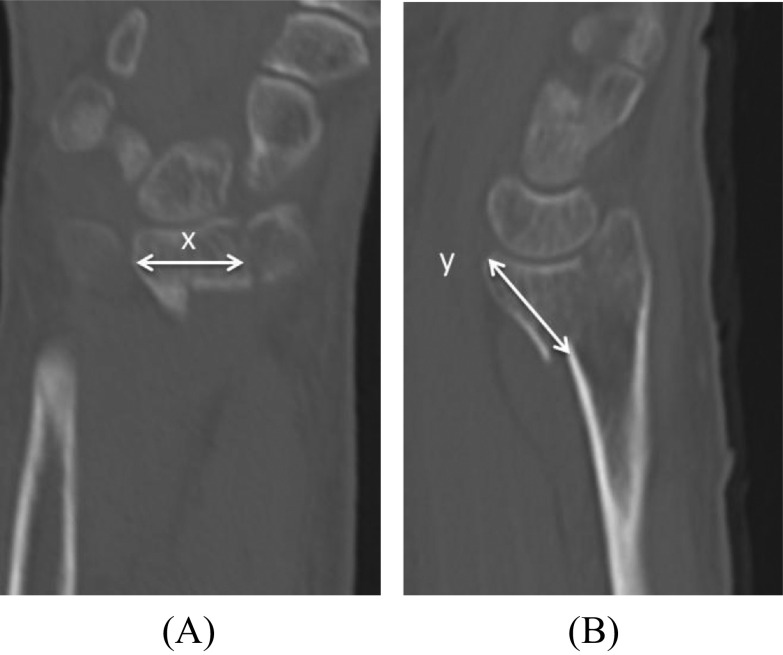
Transverse dimension of the VLF was measured where the distance from radioulnar joint to the radial aspect of fracture line of the VLF was maximum in the CT coronal plane (x) (1A). After the volar tip of the distal radius was identified in the CT axial plane the longitudinal dimension of the VLF was measured at the same point in the CT sagittal plane (y) (1B).

**Figure 2:**
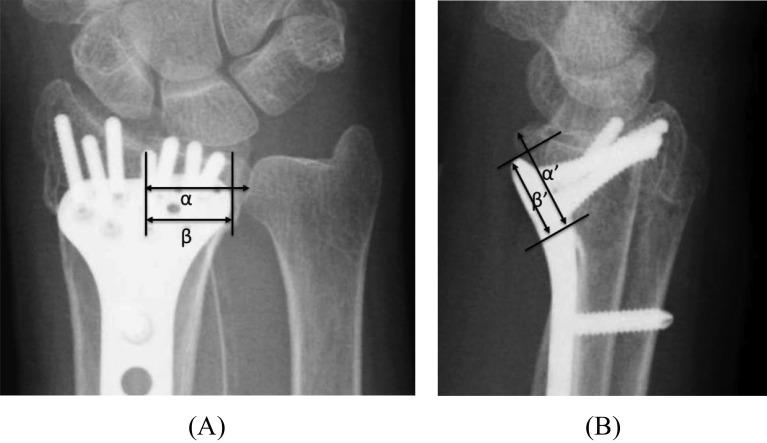
Plate coverage against the transverse dimension of the VLF was calculated as the percentage of the area covered by the plate in the transverse dimension of the VLF on AP radiographs (β/α) (2A). In the same way, plate coverage against the longitudinal dimension of the VLF was calculated as the percentage of the area covered by plate in the longitudinal dimension of the VLF on lateral radiographs (β’/α’) (2B).

Moreover, we calculated and compared longitudinal dimension and plate coverage of the VLF between the two groups to reveal the risk factors for loss of reduction of the VLF after operative fixation (Table 2). Transverse dimension of the VLF was measured where the distance from the radioulnar joint to the radial aspect of the fracture line of the VLF was maximum in the computed tomography (CT) coronal plane (Figure 1A). After the volar tip of the distal radius was identified in the CT axial plane, the longitudinal dimension of the VLF was measured at the same point in the CT sagittal plane (Figure 1B). Plate coverage of the transverse dimension of the VLF was calculated as the percentage of the length covered by the plate in the transverse dimension of the VLF on anteroposterior (AP) radiographs (Figure 2A). In the same way, plate coverage of the longitudinal dimension of the VLF was calculated as the percentage of the length covered by the plate in the longitudinal dimension of the VLF on lateral radiographs (Figure 2B). The measurement of the radiographs was conducted by an independent (non-treating) orthopedic surgeon.

**Table 2:** Radiographic characteristics.

	Group D (*n* = 10)	Group N (*n* = 25)	*p* value
Longitudinal dimension of the VLF, mm (mean [*SD*])	14.0 (5.2)	15.5 (5.1)	0.433
Plate coverage against the transverse dimension of the VLF, % (mean [*SD*])	68.5 (10.8)	81.1 (7.9)	<0.001*
Plate coverage against the longitudinal dimension of the VLF, % (mean [*SD*])	62.5 (9.3)	82.4 (11.3)	<0.001*

* Statistically significant difference.

We performed univariate analysis using Student’s *t*-test and parameters of plain radiographs and CT were compared between group D and group N. Subsequently, we performed multivariate logistic regression analysis to reveal the risk factors for loss of reduction of the VLF after operative fixation. Receiver operating characteristic (ROC) curves were generated for the risk factors and the cut-off point was determined by the area under the ROC curve (AUC). *P* < 0.05 indicated statistical significance for all analyses. All statistical analyses were performed with EZR (Saitama Medical Center, Jichi Medical University, Saitama, Japan), which is a graphical user interface for R (The R Foundation for Statistical Computing, Vienna, Austria). More precisely, it is a modified version of R commander designed to add statistical functions frequently used in biostatistics.

## Results

At final follow-up, 25 fractures maintained radiographic alignment and 10 (28.6%) lost reduction. There were nine fractures with loss of reduction before October 2015 and only one fracture with loss of reduction after October 2015.

No significant between-group differences were observed with respect to demographic variables, including age, sex, mechanism of injury, history of smoking, history of diabetes, and AO/OTA classification.

**Figure 3:**
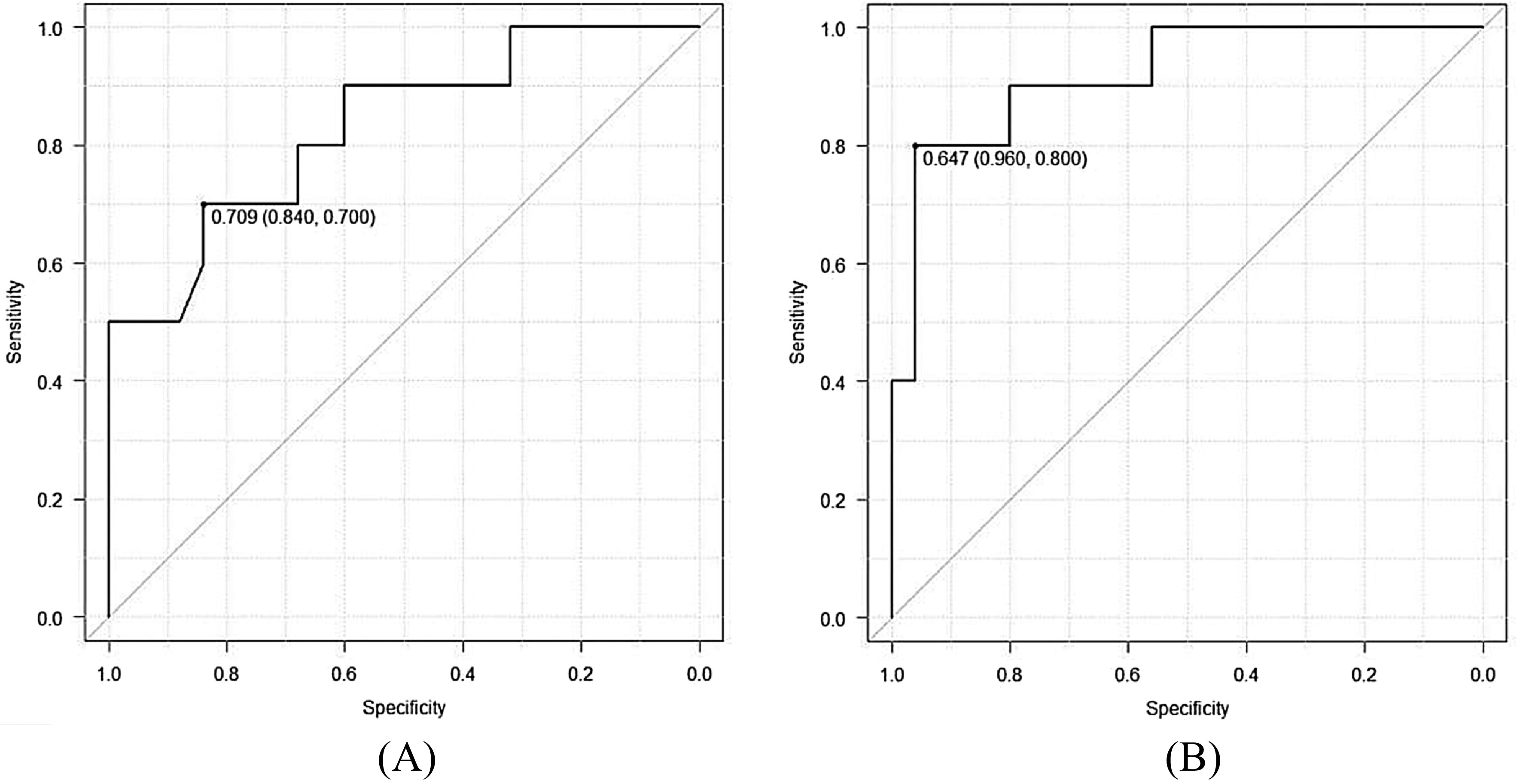
ROC curve of the transverse coverage rate of the VLF. Sensitivity and specificity were 84% and 70%, respectively (3A). ROC curve of the longitudinal coverage rate of the VLF. Sensitivity and specificity were 96% and 80%, respectively (3B).

Univariate analysis between the two groups showed that plate coverage against the transverse dimension of the VLF and plate coverage against the longitudinal dimension of the VLF were correlated with loss of reduction after operative fixation (*p* < 0.001, *p* < 0.001, respectively). In multivariate logistic regression analysis for plate coverage against the transverse and longitudinal dimension of the VLF, after controlling for age, sex, and longitudinal dimension of the VLF, only plate coverage against the longitudinal dimension of the VLF remained a significant predictor of failure (*p* = 0.029) (Table 3). The AUC for plate coverage against the transverse dimension of the VLF was 0.83. With 70.9% as the cut-off point, the sensitivity and specificity of the prediction were 84% and 70%, respectively. The AUC for plate coverage against the longitudinal dimension of the VLF was 0.92 (Figure 3A). With 64.7% as the cut-off point, the sensitivity and specificity of the prediction were 96% and 80%, respectively (Figure 3B).

**Table 3:** Multivariate analysis of the plate coverage rate for VLF.

	*p* value	Odds ratio	95% confidence interval
Plate coverage against the transverse dimension of the VLF	0.132	0.877	0.74–1.04
Plate coverage against the longitudinal dimension of the VLF	0.029*	0.852	0.738–0.983

* Statistically significant difference.

## Discussion

This study demonstrated that the predictor of loss of reduction of volarly unstable distal radius fractures with a VLF after operative fixation was plate coverage against the longitudinal dimension of the VLF. Taking the cut-off point into consideration, plate coverage against the longitudinal dimension of the VLF should be more than 65% to prevent failure.

Two patients did lose reduction after fixation despite their plate coverage against the longitudinal dimension of VLF being greater than 65%. In both cases, their plate coverage against the transverse dimension of the VLF was less than 71%. Although there was no significant difference between group D and Group N on multivariate logistic regression analysis of plate coverage against the transverse dimension of the VLF, there was a significant difference on univariate analysis. Thus, some patients likely lost reduction because of a deficiency in plate coverage against the transverse dimension of the VLF. Therefore, we recommend plates be placed as distal and ulnar as possible.

Beck et al. reported that patients with volar shearing distal radius fractures with less than 15 mm of lunate facet available for fixation, or greater than five mm of initial lunate subsidence, are at risk of loss of reduction after fixation even if a standard volar plate is properly placed [2]. In their study, greater than five mm of initial lunate subsidence was significantly correlated with loss of reduction after operative fixation in univariate analysis, but was not significantly correlated in multivariate logistic regression analysis. Therefore, we did not include it in our survey items. Proximal placement of volar locking plates entails placing the implants proximal to the watershed line. In most cases, the distance from the watershed line to the radiocarpal joint is approximately 5 mm. When the longitudinal dimension of the VLF is 15 mm, plate coverage against the longitudinal dimension of the VLF is 67% if the distal end of the proximally placed volar locking plate is placed at the watershed line. The smaller the absolute value of the longitudinal dimension of the VLF, the lower the plate coverage against the longitudinal dimension becomes. The plate coverage was less than 65% in most cases when we used proximally placed volar locking plates in patients where the longitudinal dimension of the VLF was only 15 mm. Based on this, the 65% plate coverage of the longitudinal dimension of the VLF that we reported is comparable to the risk factor of 15 mm of the VLF available that Beck reported.

The watershed line is a transverse ridge that lies distal to the pronator quadratus muscle and is an anatomic boundary point beyond which placement of volar plates risks flexor tendon irritation or rupture. If the plate has to be seated more distally to the watershed line, early removal of the plate or warning symptoms of flexor tendon irritation can reduce the risk of this complication [6]. In this study, we removed plates early for distally placed plate routinely and there was no flexor tendon rapture case.

Nowadays, several fixation options have been described for volarly unstable distal radius fractures with a small VLF to prevent loss of reduction after operative fixation, and they include a distally placed volar locking plate, a volar rim plate, fragment-specific fixation (wire form or hook plate), or the use of supplemental fixation methods such as sutures, pinning, or external fixation [4, 7–18]. These alternative fixation methods such as a distally placed volar locking plate, a volar rim plate and fragment – specific hook plate seem effective because they can increase plate coverage of VLF.

In this study, we focused on only dimension and plate coverage of VLF. But there is a possibility that the number of screws which are inserted to VLF is related to loss of reduction. Further investigations are needed.

There were several limitations of the current study. First, because our study was retrospective, multiple surgeons performed the fixation procedures. Second, we used both proximally and distally placed volar locking plates, which have different fixation concepts, and we changed the therapeutic strategy at certain times due to the findings of the study by Beck et al. It is not easy to conduct a prospective comparative study after this because the concept that the plate should be placed at the distal and ulnar side of the VLF is widely accepted, and there have been various studies discussing the prevention of loss of reduction after fixation.

## Conclusion

This study demonstrated that the predictor of loss of reduction of volarly unstable distal radius fractures with a VLF after operative fixation was plate coverage against the longitudinal dimension of the VLF. Taking the cut-off point into consideration, plate coverage against the longitudinal dimension of the VLF should be more than 65% to prevent failure. If the adequate plate coverage is not achieved by the volar locking plate, we should consider the additional fixation.

## Conflict of interest

The author declares no conflict of interest in relation with this paper.
